# Insanity from Solitary and Separate Confinement. Also, Mortality in Lunatic Asylums

**Published:** 1851-04-01

**Authors:** 


### INSANITY FROM SOLITARY AND SEPARATE CONFINEMENT. ALSO, MORTALITY IN LUNATIC ASYLUMS.

In an elaborate paper, published by Dr. Webster, in the December number of the "London Journal of Medicine," respecting the health of the metropolis during the six months terminating September 28, 1850, amongst other points of interest, allusion is made to the effect produced upon the mental faculties of prisoners confined in the various jails of London. Considering data of this description are of importance to the psychological physician, and to every person occupied in treating the insane, as also to the philanthropist, we copy from Dr. Webster's paper the following instructive paragraph; as it cannot be otherwise than interesting to our readers:

"One remarkable feature ought to be here especially mentioned, in reference to the metropolitan prisons. However beneficial confinement in such places may really prove to the bodily health of the inmates, it sometimes appears to produce an opposite effect upon their mental condition; particularly in those undergoing solitary or separate punishment. This baneful influence is fully established by the fact, that from two London prisons—viz., Pentonville and Millbank, where only convicted criminals are confined, 61 prisoners were sent to Bethlehem Hospital during the last ten years, who had become insane; 47 being men, and 14 women; besides four men who came from the hulks, but had previously resided in Pentonville prison. In addition to the above sixty-five individuals, several male and female prisoners have been also admitted from other gaols as lunatics into Bethlehem hospital, although to a much smaller extent. The effect of confinement in prison upon the mental faculties, is hence very decided; and it should be remembered, that the above sixty-five cases of insanity recently sent from the metropolitan prisons, and now reported, were not persons acquitted because they were insane, but prisoners actually undergoing sentence for previous crimes and misdemeanors."

Another quotation from the same document also deserves a place in our pages; seeing it bears upon a subject which equally comes within our province—namely, the recent mortality met with in metropolitan lunatic asylums. In reference to this point, Dr. Webster makes the following remarks, which we likewise consider well worth recording in the "Psychological Journal."

"During the last six months, the deaths in the metropolitan asylums for the insane, whose average population is about 3,527, consisting of 1,513 male, and 1,954 female lunatics, amounted to 171; 96 men, and 75 women; being one death in nearly every 16 male, and only one in every 26 female lunatics, during the last half year. This seems a moderate rate of mortality, especially amongst the female lunatics, who are also the most numerous. These facts are the more interesting, as they prove the correctness of an observation which I have elsewhere made, and which was based upon extensive data, that mania, although more common among women, is in them more curable and less fatal than among men. Female lunatics, moreover, are likely to attain greater longevity than male lunatics."

Unwilling to extend this notice to any length, however easy it would be to enlarge upon such interesting topics, we will only at present remark, that few subjects are so important as the treatment of criminals, with a view to their proper punishment and reformation, as also the effect which confinement in jails produces upon the bodily health, and, still more, the mental condition of prisoners. Hence, we have copied verbatim the above two paragraphs; and would again especially direct attention to the first, which seems to show very clearly the baneful influence of solitary confinement upon the human mind; whilst the above recorded facts strongly illustrate this very important question.

